# Giant chylous mesenteric cyst in a young adult: A case report

**DOI:** 10.1016/j.ijscr.2025.111003

**Published:** 2025-01-31

**Authors:** Sileshi Genetu Tiruneh, Serkadis Muluye Fetene, Eneyew Mebratu Gashey

**Affiliations:** a Department of Surgery, GAMBY General Teaching Hospital, Bahir Dar, Ethiopia; b Department of Pathology, GAMBY General teaching Hospital, Bahir Dar, Ethiopia

**Keywords:** Chylous, Mesenteric cyst, Transverse mesocolon

## Abstract

**Introduction:**

Mesenteric cysts are rare benign intra-abdominal lesions located in the mesentery of the gastrointestinal tract and may extend from the base of the mesentery into the retro-peritoneum. Majority of mesenteric cysts occurred in the small bowel mesentery.

**Case presentation:**

A 35-year-old male presented with a one year history of nonspecific abdominal pain and distension. Abdominal ultrasound revealed a large cystic mass. During surgical exploration, a giant mesenteric cyst was identified. Complete surgical enucleation of the cyst was successfully performed without requiring bowel resection.

**Discussion:**

A mesenteric cyst is any cystic lesion found within the mesentery. While its precise cause remains uncertain, various theories have been suggested. The clinical presentation of mesenteric cysts is variable. Surgical intervention remains the primary treatment approach for managing mesenteric cysts.

**Conclusion:**

Chylous mesenteric cysts are rare, benign intra-abdominal tumors that often present with nonspecific clinical features, making their diagnosis challenging. These cysts should always be included in the differential diagnosis when evaluating abdominal cystic lesions.

## Highlights

• Mesenteric cysts are rare benign intra-abdominal lesions.
• Preoperative diagnosis is difficult due to their noncharacteristic clinical presentation.
• Surgical intervention remains the primary treatment approach for managing mesenteric cysts.

## 1 Introduction

Mesenteric cysts are rare usually benign intra-abdominal lesions. It can arise from anywhere in the mesentery from the duodenum to the rectum [1,2]. The majority (60 %) of mesenteric cysts occurred in the small bowel mesentery, the remaining 24 % in the large bowel mesentery, and 14.5 % in the retro-peritoneum [3]. Chylous mesenteric cysts account for 7.3 % of all abdominal cysts and it was first described by von Rokitansky in 1842 [3].

Clinical manifestation ranges from asymptomatic patients to severely ill patients with peritonitis, perforation and death. Patients generally present with vague abdominal pain sometimes associated with abdominal swelling. Ten percent of patients with such cysts present as an acute abdomen [2,3]. The mainstay of therapy is complete surgical removal of the cyst [3].

In this report, we present a case of a giant chylous mesenteric cyst which was successfully managed by open surgical enucleation.

This work has been reported in line with the SCARE criteria [4].

## 2 Case presentation

A 35-year-old male presented to our hospital with a one year history of abdominal swelling and pain. He initially visited a nearby primary health facility and received unspecified medications with no improvement. A month before his presentation, he noticed a rapid increment in the size of the swelling and pain, which prompted him to visit our hospital. Despite having a good appetite, he reported early satiety. There was no history of trauma or previous abdominal surgery. He has no known chronic medical illness.

On physical examination, the patient had normal vital signs. Abdominal assessment revealed a distended, tense abdomen with dullness on percussion and a positive fluid thrill.

Laboratory tests were within normal limits. Abdominal ultrasound showed a well-defined intra-peritoneal cystic mass measuring 23 × 27 cm. A computed tomography (CT) scan could have provided a detailed evaluation of the cyst and its relationship with the surrounding structures, aiding in preoperative planning. Further investigations were not performed due to financial reasons and exploration was decided.

**Fig. 1:**
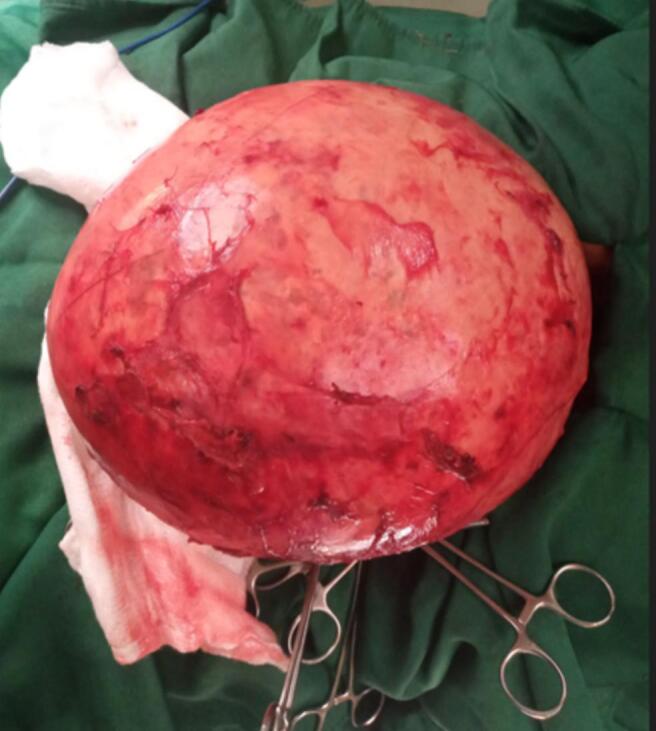
Intraoperative view of the mass.

The patient was prepared for surgery and an exploratory laparotomy was performed. Intraoperative findings included a massive cystic mass occupying nearly the entire abdominal cavity, with adhesions to the anterior abdominal wall, distal ileum, and transverse mesocolon. The mass originated from the mesentery of the transverse colon. The cyst was carefully delivered and dissected from the mesentery while preserving the blood supply (Fig. 1). The mass was successfully enucleated, and the abdomen was closed in layers.

**Fig. 2:**
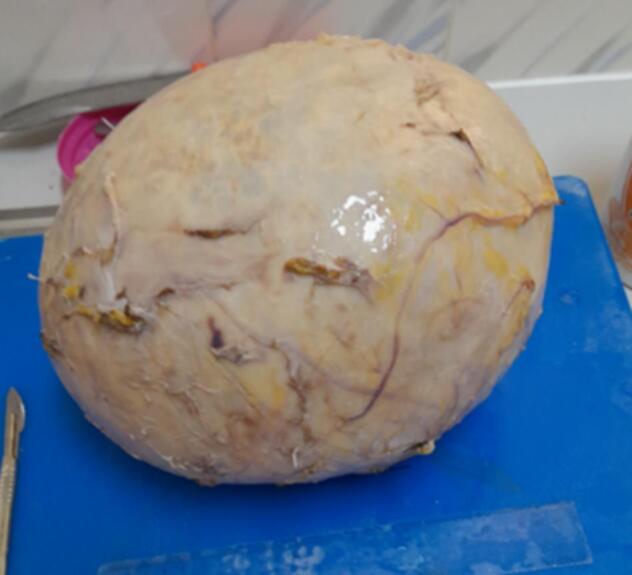
Pathologic specimen of the cyst.

**Fig. 3:**
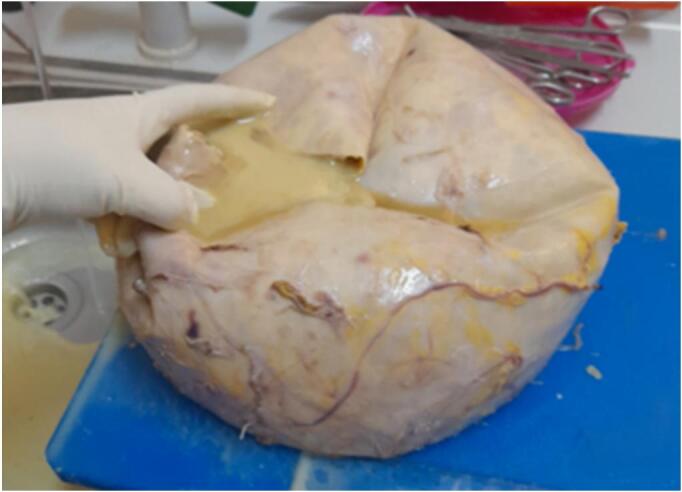
Surgical specimen of the cyst after opening it.

**Fig. 4:**
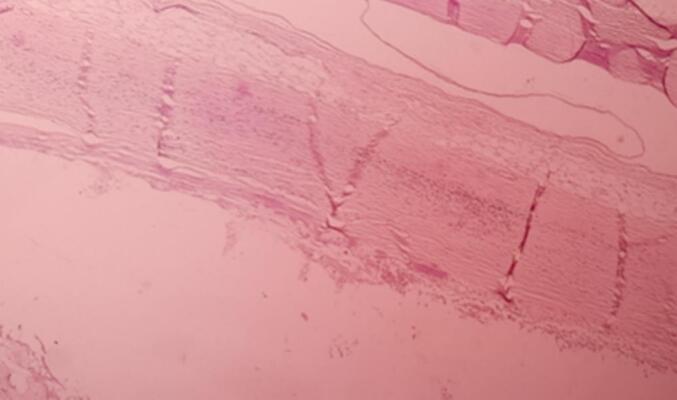
Low power examination shows cyst wall fragment.

**Fig. 5:**
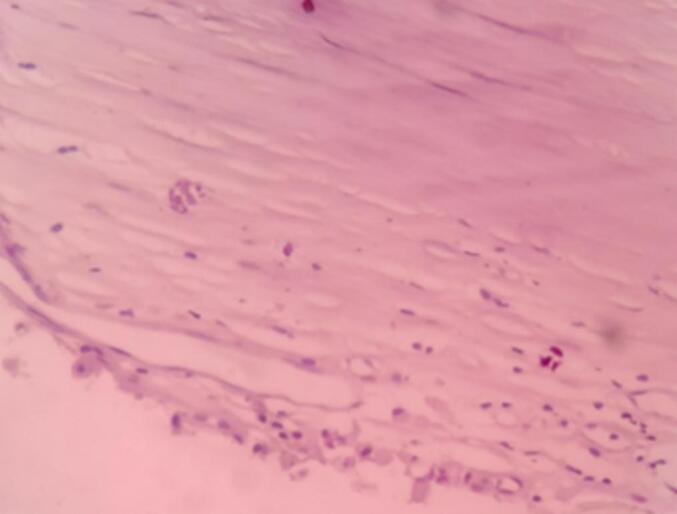
High power examination shows cyst wall lined by bland cuboidal and flattened epithelial cells.

The gross examination revealed a cystic, gray-white tissue measuring 26 × 22 × 18 cm (Fig. 2). Upon sectioning, it appeared as a unilocular cavity filled with thick, whitish fluid (Fig. 3). Histopathological analysis showed fragments of the cyst wall composed of a thick fibrous layer with areas of calcification. The cyst lining was formed by bland mesothelial cells, accompanied by focal lymphocytic aggregates (Fig. 4, Fig. 5). No evidence of atypia, malignancy, or a muscular layer was identified. Based on these findings, the diagnosis of a chylous mesenteric cyst was established.

The patient had an uneventful postoperative recovery and was discharged on the third postoperative day. Follow-up visits at one month and one year revealed no complaints or signs of recurrence.

## 3 Discussion

A mesenteric cyst is defined as any cyst located in the mesentery. These cysts are exceedingly rare, with an estimated incidence of 1 in 100,000 in adults and 1 in 20,000 in pediatric hospital admissions. Benevenni, an Italian anatomist, was the first to describe this entity in 1507 [6]. He illustrated this abdominal finding while performing an autopsy on an 8-year-old boy. Tillaux achieved the first successful surgical excision of a cystic mass in the mesentery in 1880 [1,5].

Most cysts are single but can be either unilocular or multilocular. They vary in size and shape, ranging from a few centimeters to a size that can occupy most of the peritoneal cavity as seen in our case [2].

Based on histopathological features, mesenteric cysts can be classified into six groups: (1) cysts of lymphatic origin, which include simple lymphatic cysts and lymphangiomas; (2) cysts of mesothelial origin, such as simple mesothelial cysts, benign cystic mesotheliomas, and malignant cystic mesotheliomas; (3) cysts of enteric origin, including enteric cysts and enteric duplication cysts; (4) cysts of urogenital origin; (5) mature cystic teratomas (dermoid cysts); and (6) pseudocysts, which encompass infectious and traumatic cysts [11]. The histologic findings in our patient are consistent with a simple mesothelial cyst.

The exact etiology of mesenteric cysts remains unclear, though several theories have been proposed. These theories include the continuous growth of congenitally malformed or misplaced lymphatic tissue, trauma-induced changes, degeneration of lymph nodes, or improper fusion of the mesentery's leaves during development [2].

The clinical features of mesenteric cysts include abdominal distension, vague abdominal pain, the presence of a palpable mass, intestinal obstruction, and obstructive uropathy. Symptoms arise due to compression of adjacent structures, stretching of the mesentery by rapid expansion, infection, or rupture with hemorrhage. Some cases are discovered incidentally during other surgical procedures. Malignant transformation is estimated to occur in approximately 3 %. Achieving a specific diagnosis prior to surgery is challenging due to lack of pathognomonic symptoms or imaging findings [7,9].

Diagnosing Chylous cysts can be difficult as they often mimic other intra-abdominal cystic lesions. Preoperative diagnosis can be achieved using imaging modalities such as ultrasonography, computed tomography (CT), or magnetic resonance imaging (MRI). Ultrasonography is the recommended first-line technique, as it helps localize the cystic mass and assess its relationship with nearby anatomical structures. CT scans confirm ultrasonography findings and are crucial for planning an appropriate surgical approach [8].

Surgical intervention remains the cornerstone of treatment for mesenteric cysts. The preferred approach is complete enucleation, either via open surgery or minimally invasive laparoscopic techniques. In cases where the cyst is densely adherent to adjacent tissues or organs, en bloc resection may be necessary, which could involve the removal of segments of the bowel, spleen, or pancreas. Approximately one-third of patients require bowel resection during treatment.

Less invasive procedures such as aspiration and marsupialization are associated with high recurrence and infection rates and are therefore not recommended [1,2,9]. A conservative approach with follow-up can be sufficient for asymptomatic, small cysts with no adjacent structure compression or mass effect [8].

Regular follow-ups with abdominal ultrasounds are recommended for these patients to facilitate early detection of mesenteric cyst recurrence and minimize associated morbidity. If the cyst recurs, CT-guided aspiration should be considered [10].

## 4 Conclusion

Chylous mesenteric cysts are rare, benign intra-abdominal tumors that often present with nonspecific clinical features, making their diagnosis challenging. These cysts should always be included in the differential diagnosis when evaluating abdominal cystic lesions. Early surgical intervention not only alleviates symptoms but also prevents potential complications such as infection, rupture, or intestinal obstruction.

## Abbreviations

CT: computed tomography
MRI: magnetic resonance imaging

## Author contribution

Sileshi Genetu Tiruneh: conceptualisation, writing and editing, patient management.

Serkadis Muluye Fetene: drafting of the manuscript and editing.

Eneyew Mebratu Gashey: drafting of the article and editing.

## Consent

Written informed consent was obtained from the patient for publication of this case report and accompanying images. A copy of the written consent is available for review by the Editor-in-Chief of this journal on request.

## Ethical approval

Ethical approval for this study was provided by ethical committee of GAMBY General Hospital with Ref. No GM/1050/17.

## Guarantor

Sileshi Genetu.

## Research registration number

Not applicable.

## Funding

The authors received no financial support for writing or publication of this article.

## Conflict of interest statement

We declared that there is no conflict of interest.
